# Understanding the origin of photoelectrode performance enhancement by probing surface kinetics†
†Electronic supplementary information (ESI) available: Calculations of Gärtner currents, *JV* curves of films, further IMPS data. See DOI: 10.1039/c5sc04519c


**DOI:** 10.1039/c5sc04519c

**Published:** 2016-02-11

**Authors:** James E. Thorne, Ji-Wook Jang, Erik Y. Liu, Dunwei Wang

**Affiliations:** a Department of Chemistry , Boston College , Merkert Chemistry Center , 2609 Beacon St. , Chestnut Hill , MA 02467 , USA . Email: dunwei.wang@bc.edu

## Abstract

Photoelectrochemical (PEC) water splitting holds the potential to meet the challenges associated with the intermittent nature of sunlight. Catalysts have often been shown to improve the performance of PEC water splitting, but their working mechanisms are not well understood. Using intensity modulated photocurrent spectroscopy (IMPS), we determined the rate constants of water oxidation and recombination at the surface of three different hematite-based photoanodes. It was found that the best performing electrodes, in terms of photocurrent onset potential, exhibited the slowest water oxidation rate constants, which was a surprise. The performance of these photoelectrodes was enabled by the slow surface recombination. When amorphous NiFeO_*x*_, a water oxidation catalyst, was present, the rate of surface hole transfer actually slowed down; what was slowed more was the recombination rate at the hematite surface, resulting in better water oxidation performance. As such, NiFeO_*x*_ primarily serves as a passivation layer rather than a catalytic layer. Together a better understanding of the role of catalytic overlayers for water oxidation has been achieved.

## Abstract 2

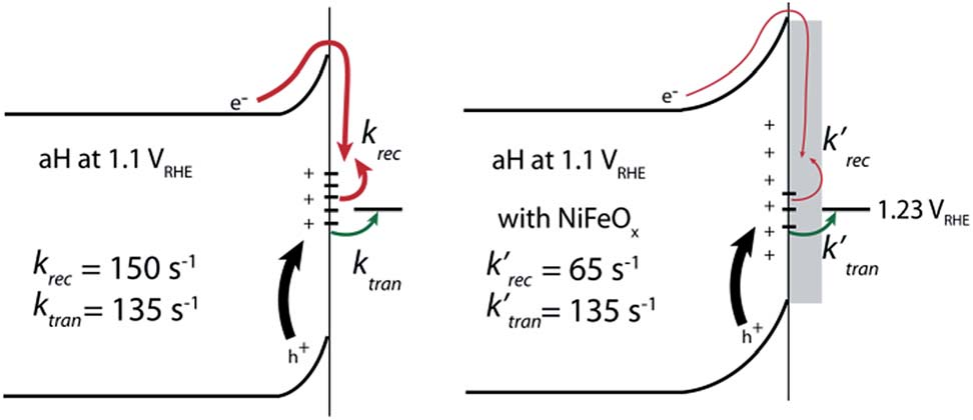
Probing the surface kinetics of different hematite electrodes with and without surface passivations.

## Introduction

Solar water splitting is an important first step in photosynthesis. Plants utilize the harvested solar energy to extract electrons from H_2_O, providing enough thermodynamic energy to reduce carbon dioxide during the dark reactions.^1^ Given the vast availability of H_2_O and sunlight, solar water splitting holds great potential for direct solar energy conversion and storage.^2^ For complete water splitting, two fundamentally important processes, the reduction reaction and the oxidation reaction, need to be balanced. Due to the complex 4-electron/4-proton nature, the water oxidation reaction tends to be more difficult and slower than the reduction reaction. This highlights the necessity for the addition of catalysts to facilitate hole transfer to H_2_O for O_2_ evolution.^3^ A number of materials, including IrO_*x*_, CoO_*x*_, RuO_*x*_, NiFeO_*x*_, Co–phosphate, NiOOH, and more recently a molecular heterogenized Ir, have been confirmed as active catalysts toward the O_2_ evolution reaction (OER).^3^–^10^ However, when integrated with light absorbers, the role of these catalysts is often a source of confusion and debate (*vide infra*). This is because the presence of catalysts on a photoelectrode surface not only changes the charge transfer kinetics, but they can also alter the surface energetics that define the charge separation capabilities near the photoelectrode surface.^11^ Further development of the water splitting field will require a detailed understanding of the mechanisms that underpin the observed performance, which in turn depends on the quantitative measurement of surface energies and charge transfer kinetics. Using hematite as a prototypical photoelectrode system, we show that surface modifications of hematite by either a re-growth strategy or with an amorphous NiFeO_*x*_ catalyst serve to reduce surface recombination. Whereas, the charge transfer kinetics are either slower or remain unchanged for all scenarios studied here, strongly supporting that a surface passivation effect is the key reason for the observed improvement in water oxidation by hematite.

Hematite was chosen as the material for the present study as it has been at the center of a recent debate about how catalysts alter the photoanodic characteristics of water oxidation.^12^,^13^ Despite the appeals of low cost and good stability, hematite faces great challenges as a photoelectrode material due to its low photovoltages and poor catalytic activities.^14^–^16^ The slow hole transfer kinetics across the hematite/water interface, in particular, has been considered a key reason for the low photovoltages.^17^,^18^ Therefore there have been numerous attempts targeted at reducing the kinetic overpotentials in hopes to overcome the issues connected to the slow surface kinetics and improve the onset potential to more cathodic potentials. For example, Grätzel *et al.* applied Co^II^ onto hematite's surface and observed appreciable cathodic shift of the steady-state current–voltage relationship (∼80 mV), which was thought as a direct proof of how fast hole transfer improves the photoelectrochemical (PEC) performance.^19^ A more pronounced effect was observed by Gamelin *et al.* with a Co–phosphate OER catalyst (Co–Pi; 200 mV),^20^ with similar phenomena having been reported on other catalyst/photoelectrode combinations.^21^–^23^ However, the view that the change in the PEC performance was due to better charge transfer was challenged by Durrant *et al.*'s transient absorption measurements, where longer surface hole lifetimes, rather than faster hole transfer kinetics, were concluded to be the reason for the cathodic shift.^24^ Subsequent detailed electrochemical studies by Hamann and Bisquert *et al.* suggested that Co–Pi on hematite may serve dual functionalities of promoting charge transfer and building up hole concentrations.^25^ Their results, nevertheless, do not fully settle the debate as a quantitative understanding of the system is still missing. In parallel, our group has conducted studies on combining amorphous NiFeO_*x*_ with hematite and attributed the cathodic shift in onset potential to better charge separation.^26^,^27^ Our key experimental evidence comes from the measurement of the surface energies of the system under equilibrium or pseudo-equilibrium conditions. With the addition of a-NiFeO_*x*_ to the surface of our regrown hematite samples, we have achieved the best turn on potential observed for hematite (0.45 V *vs.* RHE).^28^ However, our initial studies indicate that the cathodic shift in onset potential is mostly, if not entirely, due to surface energetics rather than improved water oxidation kinetics. Again, for a complete understanding of the system, quantitative measurements of the surface charge transfer kinetics are necessary. The results reported herein fill in this knowledge gap.

On a fundamental level, the key driving force of a photoanodic reaction is the accumulation of energetic holes on the photoelectrode surfaces. While the detailed dependence of the reaction rate on the hole concentration may be intricate, as revealed by several recent studies, a high steady-state surface hole concentration is generally desired,^29^ where a higher concentration of surface holes results in higher photovoltages that help reduce the need for externally applied potentials. To the first order approximation, the rate at which the holes are generated depends on the light absorption and charge separation of the photoelectrode, as well as the light intensity. The rate at which the holes disappear depends on the rate of surface recombination and hole transfer into the solution. Note that contributions by bulk recombination may be regarded as part of the photoelectrode charge separation and is not considered here. We see from this simplified argument that, for a given system under fixed lighting conditions, detailed knowledge of surface recombination rates and charge transfer kinetics is critically important. Although the information may be inferred by probing hole concentrations using, for instance, spectroscopic methods, or by fitting electrochemical impedance data,^30^–^32^ a direct measure of these kinetic parameters is of great value. For this purpose, we employed a technique pioneered by Peter *et al.*, known as intensity modulated photocurrent spectroscopy (IMPS).^33^ In using IMPS, we are presented with an opportunity to systematically compare samples of different surfaces that feature different PEC performance. This information leads to a better understanding of the kinetics at the photoelectrode/water interface that has been missing in the literature. By examining hematite prepared by three different methods (atomic layer deposition (denoted as aH), solution synthesis (denoted as sdH), and re-growth treated (denoted as rgH); see Experimental section for details) with and without surface modifications (amorphous NiFeO_*x*_ catalysts), we show that in all cases the cathodic shift of PEC water oxidation is only driven by the suppression of surface recombination, while no contribution by faster hole transfer kinetics was observed.

**Fig. 1:**
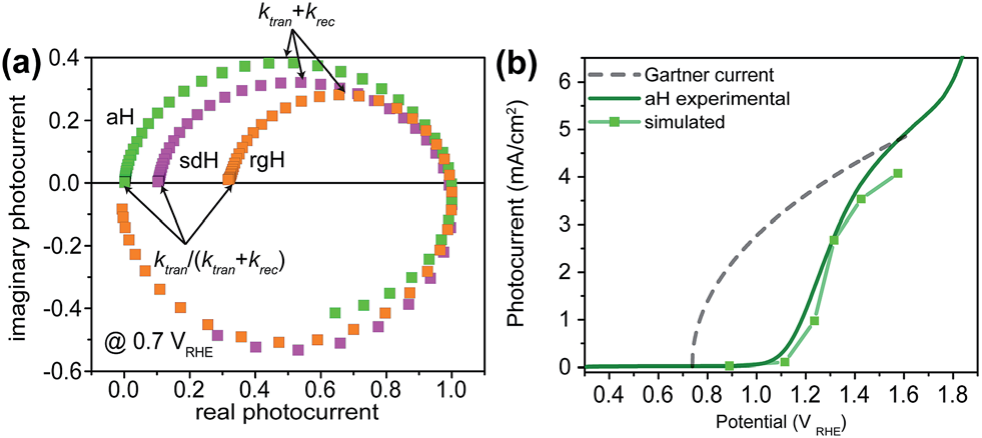
Representative IMPS and PEC data for hematite under monochromic illuminations (*λ* = 405 nm; intensity: 134 mW cm^–2^). (a) Nyquist plots of the three bare samples, aH, sdH, and rgH, at an applied potential of 0.7 V *vs.* RHE (reversible hydrogen electrode). The sample IDs are explained in the main text. The two key parts in the plots used for data analysis are highlighted by arrows. The real and imaginary photocurrents are normalized to the high frequency intercept, which represents the hole flux toward the surface. (b) Using transfer efficiency extracted from IMPS data and the Gärtner equation, we obtained the photocurrent for ALD hematite (light green between squares), which is in agreement with the experimental steady-state current–voltage curve (dark green).

IMPS is a form of impedance spectroscopy that measures the phase shift in photocurrent in relation to a sinusoidal modulation of the light source.^33^ By assuming that the small changes in the light intensity only alter surface charge concentrations but not the degree of band bending, IMPS probes how the photocurrent of a PEC system responds to these changes in light intensity. The complex photocurrent, as a function of the light modulation frequency, may be presented in a Nyquist plot (Fig. 1). In this plot, at the high-frequency intercept with the real photocurrent axis, the surface recombination rate is insufficient to match the rate at which the surface hole concentrations change as a result of light intensity modulations. This value provides a measure of the hole flux moving toward the photoelectrode surface. Note that in hematite samples the bulk recombination kinetics are much faster than the surface recombination kinetics, and a separate technique is needed in order to study bulk recombination rates. At the low frequency intercept, it reports on the steady-state photocurrent, which measures the rate at which the charges are transferred to the solution for water oxidation. The ratio of the photocurrents between the low-frequency intercept and the high-frequency one provides the charge transfer efficiency (TE), defined as *k*_tran_/(*k*_tran_ + *k*_rec_), where *k*_tran_ and *k*_rec_ are the surface charge transfer and recombination rate constants, respectively. The rate constants at the apex of the semicircle, where the maximum phase shift is measured, reports on the combined rate of charge transfer and recombination (*k*_tran_ + *k*_rec_), where the effective hole transfer from the semiconductor is the measured *k*_tran_. The theoretical basis for IMPS was developed by Peter *et al.* in a series of publications,^33^,^34^ and has been recently applied to study hematite.^35^,^36^ By extending the technique to a systematic study of a series of photoelectrodes with the same crystal structure but different surfaces, we unambiguously confirm that the passivation effect, rather than better surface catalytic activities, is the true reason for the observed PEC performance improvement. As such, the results presented here are new and significant.

## Results and discussion

### Summary of samples studied

A total of 6 types of samples were studied here. They were based on three different synthesis methods, (1) atomic layer deposition (ALD) grown hematite (aH); (2) solution derived hematite (sdH); and (3) hematite subjected to surface regrowth (rgH; note that here rgH refers to hematite that has been subjected to regrowth treatments twice). The details of their syntheses have been recently reported elsewhere and are summarized in the Experimental section.^28^ Among these samples, aH features a high degree of surface states and the highest turn-on voltages (*V*_on_, as defined by the lowest applied potential at which appreciable photocurrents are measured). The rgH samples exhibit the lowest *V*_on_'s. In addition to samples with bare surfaces, studies were performed on samples coated with amorphous NiFeO_*x*_ and GaO_*x*_.

### IMPS data and validation of the technique for the present study

A set of representative Nyquist plots for bare hematite are shown in Fig. 1a, from which the surface charge transfer efficiencies (TE = *k*_tran_/(*k*_tran_ + *k*_rec_)) were extracted. The hole flux into the photoelectrode surface can be calculated following the Gärtner relation (eqn (1)). Here the Gärtner equation is employed for its simplicity. A more complete treatment that considers the recombination in the space charge region and back flow of electrons to the electrolyte has been developed by Reichman.^37^ Intriguingly, when the charge transfer efficiency is combined with the Gärtner model, a close match with the experimental data is already obtained, raising a question as to how significant the recombination in the space charge region is in limiting the performance of hematite. Here we assume the depletion width (*W*_SC_) changes with the applied potential (*V*_app_) following, *W*_SC_ = (2*εε*_0_(*V*_app_ – *V*_fb_)/*N*_D_)^1/2^, where *ε* = 60 is the dielectric constant of hematite; *ε*_0_ is vacuum permittivity; *V*_fb_ = 0.73 V *vs.* RHE (reversible hydrogen electrode) is the flatband potential for aH; and *N*_D_ = 2.65 × 10^19^ cm^–3^ is the carrier concentration. Both *V*_fb_ and *N*_D_ were measured by the Mott–Schottky method (see ESI†). The measurable photocurrent was then obtained by combining the Gärtner hole flux with TE, as is described in eqn (1):

1 *J* = *J*_0_(1 – exp(*αW*_SC_/(1 + *αL*_p_)))TE

At *λ* = 405 nm, an absorption coefficient (*α*) of 1.0 × 10^5^ cm^–1^ was used. The hole diffusion distance of 3 nm was used, as hematite typically has a value of 2–4.^38^ Guided by our previous research, a reflection of 20% was considered for the light intensity (*J*_0_).^28^ Finally, the potential drop within the Helmholtz layer was compensated for (see ESI†). As seen in Fig. 1b, a close match between the simulated photocurrent, based on the TE obtained from the IMPS data, and the experimental data was obtained. Importantly, since all parameters have physical significance, the match was achieved without adjusting any parameters. Seeing how the TE can correct the Gärtner current for surface processes shows the power of IMPS as a tool to examine surface kinetics, and encouraged us to employ it in a systemic study of hematite's surfaces.

### Rate constants of charge transfer and surface recombination for bare hematite

**Fig. 2:**
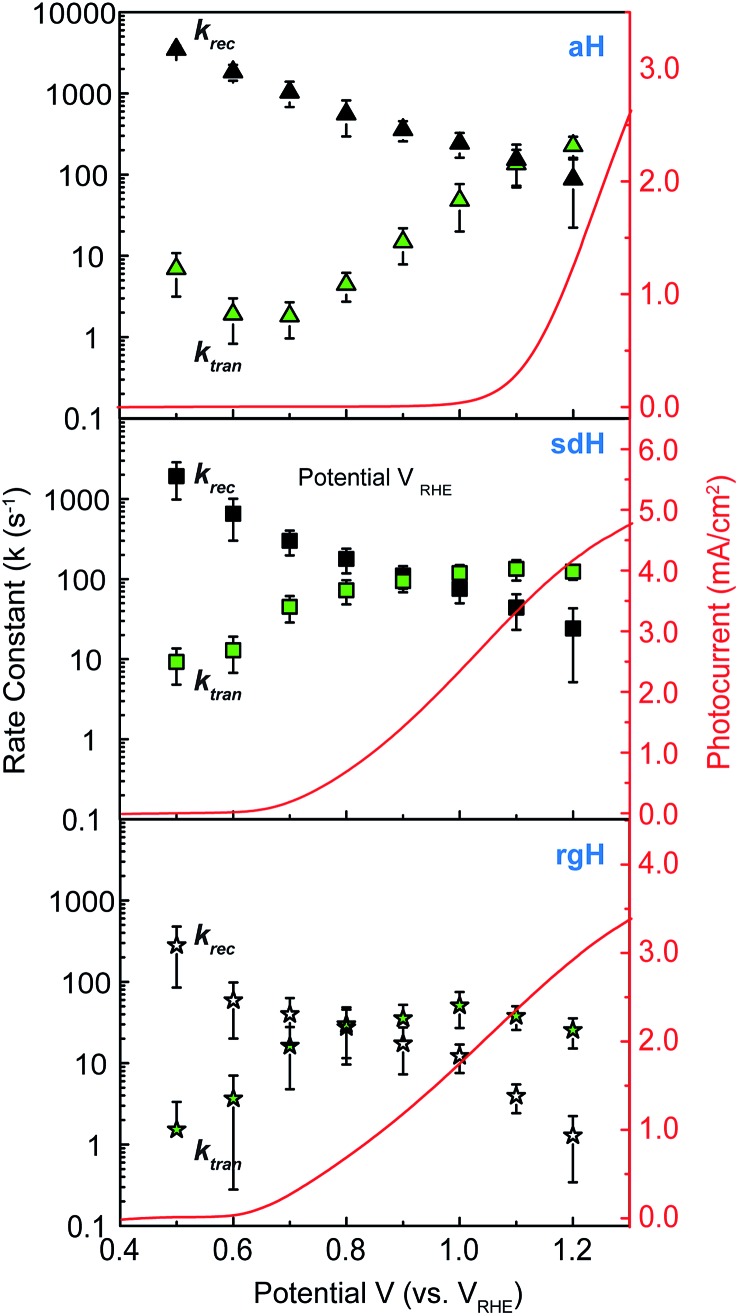
Rate constants extracted from IMPS data at different applied potentials. Top: aH; middle: sdH; and bottom: rgH. Rate constants of recombination are shown as black symbols; rate constants for surface charge transfer are shown as green symbols. The error bar is the standard deviation between different samples (typically 4 to 5 samples are measured for each group of data).

The charge transfer rate constant (*k*_tran_) and surface recombination rate constant (*k*_rec_) were obtained by combining the TE at the low-frequency intercept with *k*_tran_ + *k*_rec_, as determined by the apex of the semicircle in Fig. 1a. Note that a first order dependence of the reaction rate on the hole concentration is assumed here. The assumption is consistent with experimental results reported to date. Recently, Durrant *et al.* observed that at 1.5 V a transition from 1st to 3rd order reactions took place at high light intensities.^29^ The third order reactions would indeed make the data interpretation more difficult. Nevertheless, we have found a close correlation between our measured water oxidation rate constants with previously reported values, confirming that the water oxidation as measured in our experiments is likely first order. As shown in Fig. 2, a monotonic decrease of *k*_rec_ is observed for all three bare hematite samples studied here. Notably, the recombination rate constants across the entire potential range are much higher for aH than sdH or rgH. This trend agrees with our previous observation by X-ray absorption that greater defect densities are present on aH and lowest for rgH.^28^ The second notable feature of this set of data is the *k*_tran_ for aH is on the rising curve at *V* > *V*_on_, whereas *k*_tran_'s for sdH and rgH plateau at ∼120 s^–1^ and ∼35 s^–1^, respectively. The results suggest that the mechanisms involved in water oxidation may be different for hematite with different surfaces. This is consistent with literature reports that water oxidation by hematite is mediated by surface-adsorbed species (also denoted as “surface states”).^27^,^39^ Thirdly, *k*_rec_ for aH is considerably higher near the turn-on potential (167 s^–1^ at 1.1 V) than that for sdH (110 s^–1^ at 0.9 V), which is still higher than that for rgH (27 s^–1^ at 0.8 V). This supports that surface regrowth treatment indeed reduces surface recombination, as we previously concluded by open-circuit potential measurements.^28^ Lastly, the potential at which *k*_tran_ surpasses *k*_rec_ coincides with the *V*_on_, further proving that IMPS provides a valid measure of the surface kinetic constants.

It is further noted that for a PEC system, a higher light intensity is expected to lead to a greater degree of quasi-Fermi level splitting. The corresponding driving force for hole transfer would increase, as well. Consequently, faster charge transfer (*i.e.*, greater *k*_tran_) is expected as light intensity is increased. This trend is indeed observed in our control experiments, where *k*_tran_ increases with light intensity monotonically (Fig. S6†).

### Rate constants of hematite photoelectrodes with NiFeO_*x*_ catalysts

**Fig. 3:**
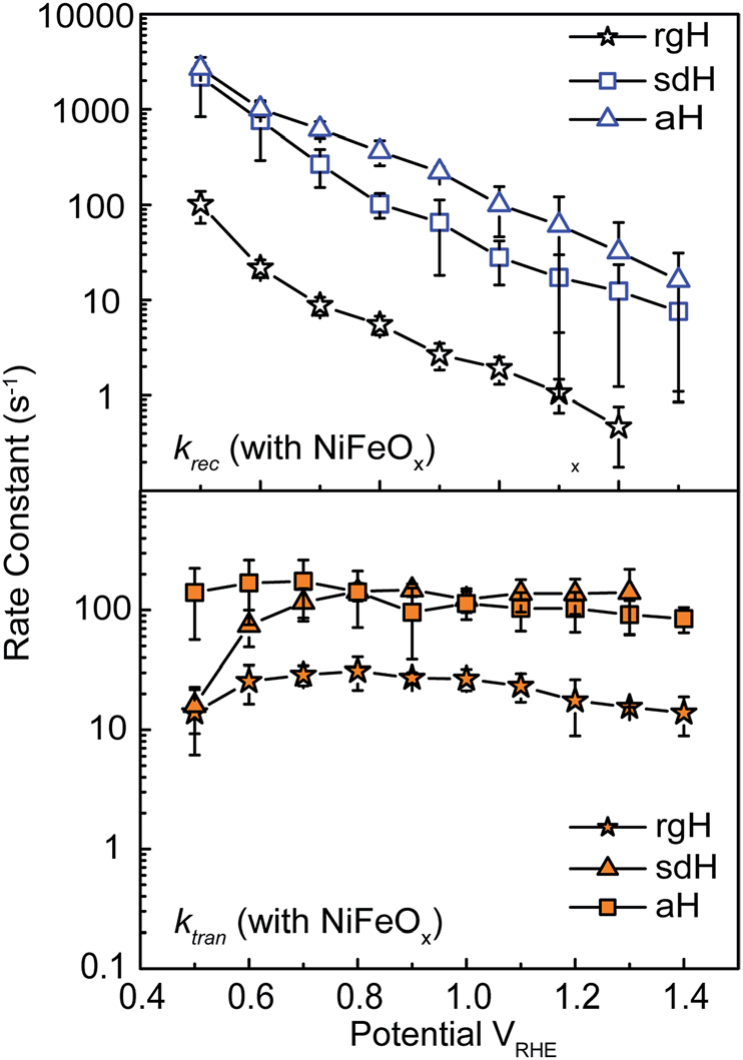
Rate constants for hematite photoelectrodes with NiFeO_*x*_ surface decorations. Top: recombination rate constants for photoelectrodes by three different preparation methods; bottom: surface charge transfer rate constants for the same photoelectrodes. The error bars are standard deviations between different samples.

With the presence of NiFeO_*x*_ as an OER catalyst, a distinctly different trend of *k*_rec_ and *k*_tran_ was observed than when compared to the bare hematite photoelectrodes. As shown in Fig. 3, *k*_tran_ appeared to be independent of *V*_app_. A similar phenomenon has been previously observed by Peter *et al.* on Co-treated hematite photoelectrodes.^35^ However, there are several features of this set of data that deserve further discussion. First, *k*_tran_'s for all photoelectrodes decorated by NiFeO_*x*_ are equal to or smaller than those without the catalyst. This is a surprise since the presence of a catalyst was expected to increase water oxidation rates. Rather, a significant suppression of the *k*_rec_'s was found, as shown in Fig. 3a. It is therefore concluded that the main function of NiFeO_*x*_ is to passivate the hematite surfaces against recombination by reducing the surface Fermi pinning, effectively increasing the band bending at the surface, rather than activating the surface for water oxidation. Note that the smaller *k*_tran_ measured here on NiFeO_*x*_ does not suggest water oxidation is slower. After all, NiFeO_*x*_ has been confirmed as a good water oxidation catalyst.^3^*k*_tran_, as measured by IMPS, likely reports on the rate determining steps (RDS) of the complex charge transfer processes from the photoelectrode to water. These results agree with Durrant *et al.* measurements of Co–Pi coated hematite photoelectrodes.^17^,^40^ They also agree with our open-circuit potential measurements.^26^,^27^,^41^ Second, the *k*_tran_'s for aH and sdH are comparable when NiFeO_*x*_ is present on the surfaces across the entire voltage range (Fig. 3a). It supports that the water oxidation processes are now governed by NiFeO_*x*_. But the *k*_tran_'s for rgH photoelectrodes are considerably lower, by almost a factor of 5. A possible reason is that the NiFeO_*x*_ employed here is *ca.* 10 nm in thickness. Its functionality may be influenced by the interface between hematite and NiFeO_*x*_. Another reason is that *k*_tran_ as measured here is the apparent charge transfer rate constant of the RDS that follows a first order dependence on the hole concentration, which may be an oversimplification of the complex water oxidation process. More research is needed to fully understand the differences. Despite rgH's slow *k*_tran_'s, the *k*_rec_'s for the rgH photoelectrodes are much slower than *k*_rec_'s for sdH or aH, by more than 10 times across the entire voltage range, further highlighting the importance of measuring photocurrent is a high TE but not necessarily a high *k*_tran_ (*vide infra*).

**Fig. 4:**
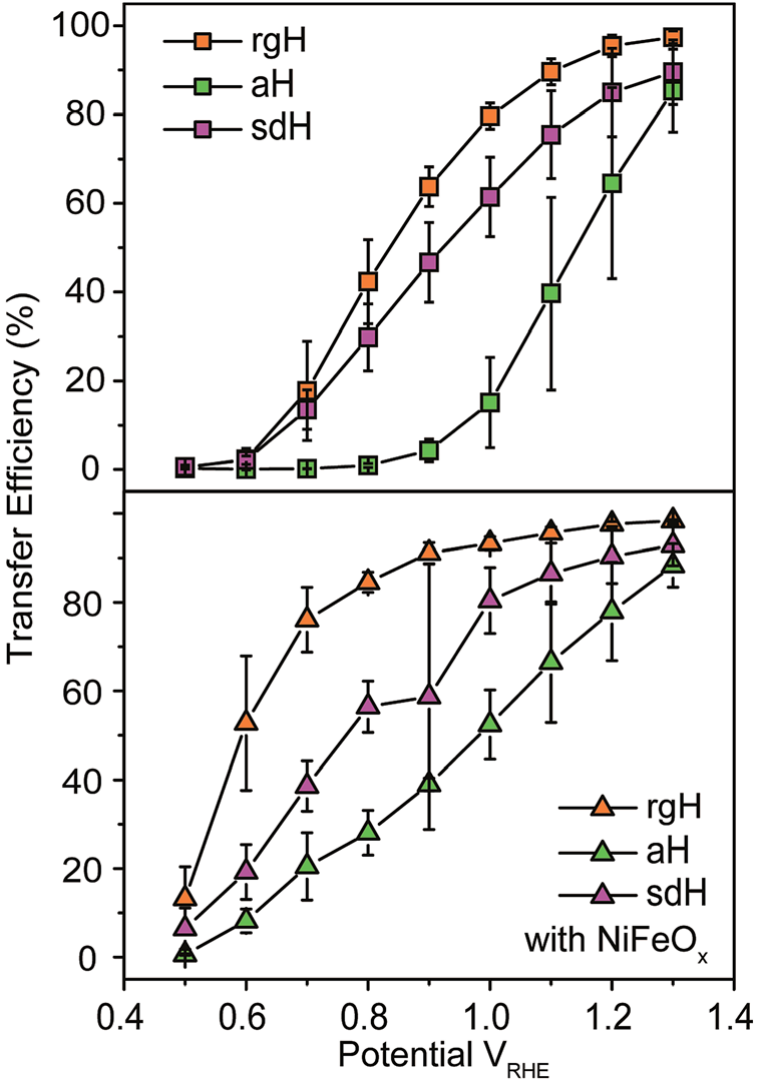
Charge transfer efficiencies for various hematite photoelectrodes without (top panel) and with (bottom panel) NiFeO_*x*_ decorations at different applied potentials.

The large decrease in *k*_rec_ led to rgH photoelectrodes having the best measured TE (Fig. 4), consistent with the observation that the best PEC performance, in terms of photovoltages and photocurrents, was measured on rgH. It highlights that as far as PEC performance is concerned, it is equally, if not more, important to consider *k*_rec_ as well as *k*_tran_, as defined by the TE. Examining eqn (1), we see that so long as the light absorption and charge separation by the photoelectrode are fixed, the determining factor for the measurable photocurrent is the TE. In other words, under the likely assumption that the main mechanisms to dissipate surface holes are charge transfer for water oxidation and surface recombination, fast water oxidation kinetics is not a prerequisite for good anodic PEC performance, so long as surface recombination can be minimized. This understanding has major implications for water oxidation reactions because, as the overall reactions are multi-electron, multi-proton in nature, they are considered inherently slow, with the characteristic rate constants in the range of millisecond (ms) or longer.^32^,^35^ Our data in Fig. 2 and 3 support such an estimate. By comparison, charge lifetimes within the bulk of semiconductors are typically in the microsecond (μs) or shorter time scales. This disparity of time scales creates a major challenge. In order to beneficially balance the effects of the solid state and water oxidation processes it is necessary to consider their resulting interactions. It is known that the thermodynamic energy of holes on the surface of a photoanode is connected to the hole concentration by: *V*_ph_ = (*k*_B_*T*/*q*)ln(*P*_s_/*P*0s), where *V*_ph_ is the photovoltage as defined by the difference between the quasi-Fermi level of holes (*E*_f,h_) and flatband potential (*V*_fb_): *V*_ph_ = *E*_f,h_ – *V*_fb_; *k*_B_ is the Boltzmann constant, *T* is the temperature, *q* is the fundamental charge of electrons, *P*_s_ is the steady-state surface hole concentration under light, and *P*0s is the equilibrium surface hole concentration under dark. It is seen that greater steady-state surface hole concentration, which is determined by the transfer efficiency, translates to higher *V*_ph_ and is desired for greater oxidation power. From this standpoint, the slow kinetic constants in both recombination and charge transfer, but more so in the slow recombination, is desired as it contributes to the build-up of surface hole concentrations, which translates to greater photovoltages. The interplay between *V*_ph_ and TE has been confirmed by our earlier work, where *V*_ph_ of 0.56 V and 0.8 V was measured on rgH without and with NiFeO_*x*_, respectively.^28^ As a comparison, only 0.24 V was measured on bare aH, whereas 0.42 V and 0.66 V was measured on sdH without and with NiFeO_*x*_, respectively.^28^

### Rate constants of hematite photoelectrodes decorated with GaO_*x*_ passivation

**Fig. 5:**
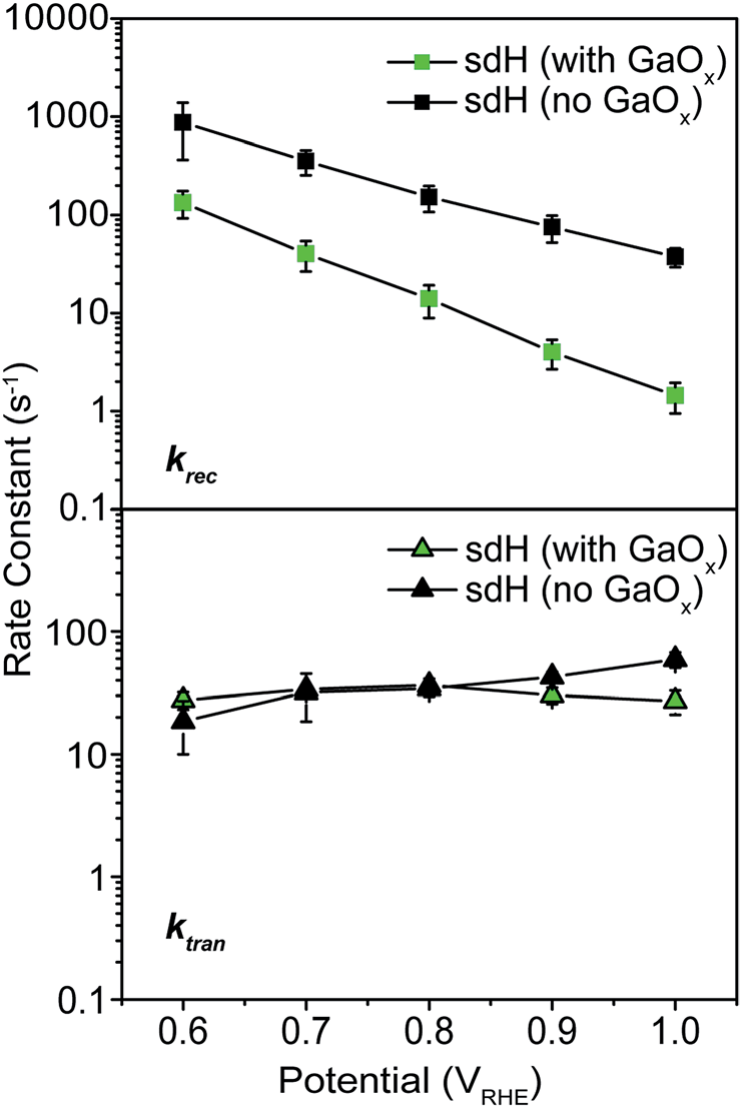
Rate constants for hematite treated with GaO_*x*_ passivation. Top: recombination; bottom: transfer.

As a control experiment, we next performed IMPS characterizations on GaO_*x*_-treated hematite photoelectrodes (sdH was studied here). GaO_*x*_ was chosen here as it has previously been shown by Grätzel *et al.* to passivate the surfaces of hematite,^42^ and Durrant *et al.* have also shown that GaO_*x*_ behaves similarly to Co–Pi on hematite when they studied the transient decay kinetics.^40^ No known catalytic effects of water oxidation were reported on GaO_*x*_. The IMPS data as shown in Fig. 5 are to be compared with those in Fig. 2 and 3. It is seen that within the range of 0.6 and 1.0 V *vs. V*_RHE_, *k*_tran_'s for both hematite with and without GaO_*x*_ only increased marginally. In contrast, *k*_rec_'s are significantly reduced due to the application of GaO_*x*_. The trend is consistent with that in Fig. 2 for bare sdH. These data further support Grätzel's proposition that GaO_*x*_ improves the PEC performance of hematite by passivating the surfaces.^42^

### IMPS studies of PEC systems with H_2_O_2_ as hole scavengers

**Fig. 6:**
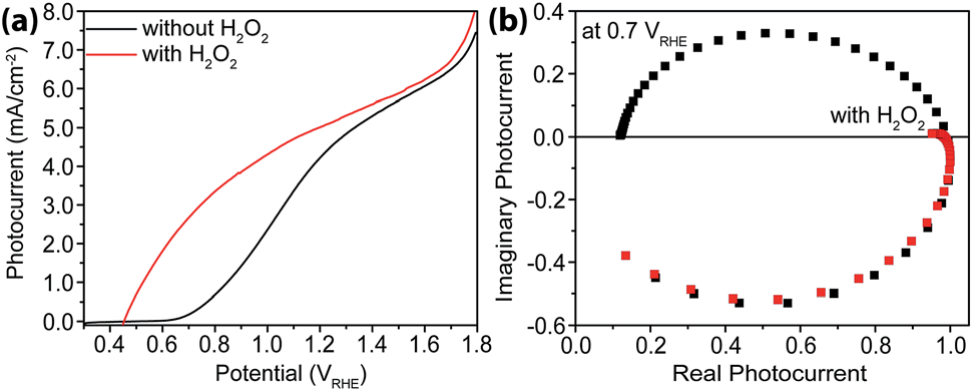
Characterizations with H_2_O_2_ as hole scavengers. (a) Steady-state current–voltage curves for sdH with (red) and without (black) H_2_O_2_. (b) IMPS data of sdH with (red) and without (black) H_2_O_2_ at 0.7 V *vs.* RHE.

As a hole scavenger that features fast charge transfer, H_2_O_2_ has been frequently used in studies on photoanodes.^43^,^44^ For instance, it is so efficient in receiving holes that in its presence the charge collection efficiency has been considered unity. Our next task was to carry out IMPS studies with H_2_O_2_ present, and the purpose was to measure charge transfer efficiencies (TE). As expected, near unity TE was obtained (Fig. 6) when 0.05 M H_2_O_2_ was added to the electrolyte. Under these conditions, because *k*_tran_ is significantly greater than *k*_rec_, the same analysis as outlined above in extracting *k*_tran_ and *k*_rec_ is no longer suitable. For this reason, no quantitative rate constant calculations were performed using IMPS.

### Intensity modulated photovoltage characterizations with and without hole scavengers

**Fig. 7:**
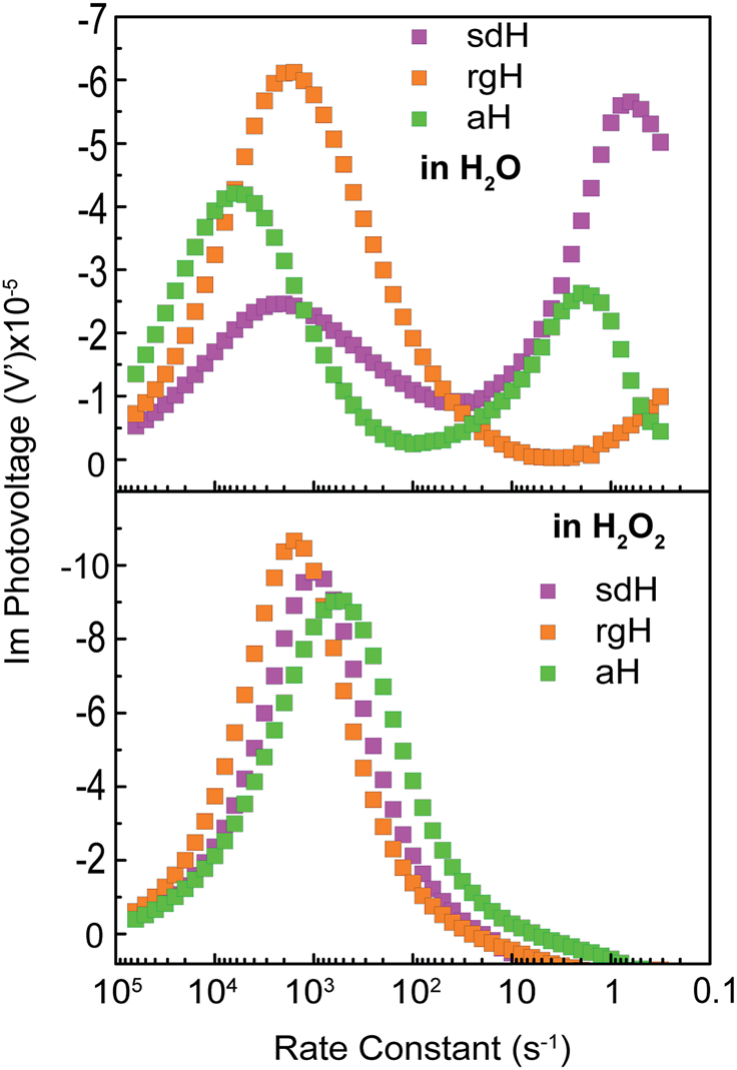
Intensity modulate photovoltage spectroscopy (IMVS) of various hematite photoelectrodes in H_2_O (top) and in H_2_O_2_ (bottom). Data presented as Bode plots, where the *Y* axis shows the imaginary component of the complex photovoltage, and the *X* axis shows the rate constants converted from the modulation frequencies.

An alternative measurement enabled by the varying light intensities is IMVS (intensity modulate photovoltage spectroscopy). Different from IMPS, IMVS probes the how the photovoltage changes as a function of the frequency of the perturbation to the light intensity under open circuit conditions. Since the external circuit is open, the net exchange current is expected to be zero. Changes in the surface hole concentration as probed by the photovoltage are expected to chiefly respond to surface recombination. When presented in Bode plots (Fig. 7), the peak frequencies report on the characteristic rate constants of surface charge recombination. In the absence of H_2_O_2_, two peaks are observed (top panel, Fig. 7). The first peak, at the higher rate constant, corresponds to a rate constant of approximately 1000 s^–1^ or faster for all three electrodes studied. When understood as surface recombination rate constants, these values agree with those obtained by analyzing IMPS data (Fig. 2). The corresponding processes are likely connected to states due to H_2_O adsorption onto hematite. This agrees with our previous research that these states are due to chemical adsorption rather than structural defects.^27^ It is also consistent with a recent EIS study by Hamann *et al.* on hematite for methanol oxidation.^39^ That the peak positions by and large remain the same for all three types of hematite electrodes further supports this understanding. Furthermore, the addition of H_2_O_2_ does not shift these peaks (Fig. 7, bottom panel) because the majority chemicals are still H_2_O in the latter case. It is noted that the measured steady-state open circuit potentials under the same lighting conditions for this set of experiments were 0.53 V, 0.52 V, and 0.49 V (*vs. V*_RHE_) for aH, sdH, and rgH, respectively. These values need to be taken into account when comparing the rate constants obtained in Fig. 7 and with those in Fig. 2.

The second peak, only observed in the absence of H_2_O_2_, appears at much lower frequencies. At 5 s^–1^ or slower, this rate constant likely corresponds to recombination by states directly connected to water oxidation. Under open circuit conditions, although the net exchange current is zero, charge transfer to oxidize H_2_O still takes places, albeit in a transient fashion as a continued H_2_O oxidation cannot be sustained. That is, the transferred charges will be annihilated by processes akin to back electron transfer that has been observed in studies on dye-sensitized solar cells.^45^,^46^ As such, it is reasonable to understand the second rate constants as water oxidation kinetics. There are two pieces of evidence that support this understanding. First, the peak rate constant for aH is the highest, followed by that of sdH. The peak for rgH is still developing, and the corresponding rate constants are likely slower than 0.1 s^–1^. The trend and the rate constants quasi-quantitatively agree with those presented in Fig. 2. Second, while the first group of peaks remains unchanged in the presence of H_2_O_2_, the second group disappeared completely, supporting that the second peaks are indeed connected to water oxidation (but not H_2_O_2_ oxidation). Taken as a whole, we understand the IMVS data as an additional proof that measurements under modulated lighting conditions can provide valuable surface kinetic information.

## Conclusions

**Fig. 8:**
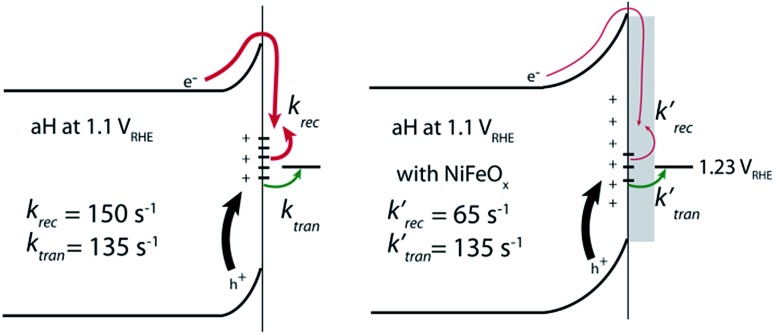
Surface decorations on hematite improves the overall PEC performance by reducing surface recombination. Compared with bare hematite (left), NiFeO_*x*_ does not change *k*_tran_ (right). Instead, it reduces the recombination rate constant (*k*_rec_) greatly.

In conclusion, we measured the surface charge recombination and transfer rate constants in a quantitative fashion. As summarized in Fig. 8, our key results are that the regrowth procedures and surface decorations by NiFeO_*x*_ OER catalyst improve the PEC performance by reducing surface recombination. Charge transfer rate constants remained unchanged for all samples studied. It was understood that slower surface recombination rates help to increase surface hole concentrations, which is measured as greater surface photovoltages. Together, with a higher charge transfer efficiency, more holes with higher energies are transferred to the solution for water oxidation. As such, for all surface decorations studied here, a passivation effect prevails. We envision that while maintaining a slow surface recombination rate, faster charge transfer should indeed lead to even better PEC performance.

## Experimental

### Hematite preparation

For the sdH and rgH syntheses, β-FeOOH was first grown on fluorine-doped tin oxide (FTO) substrates (∼7 Ω sq^–1^, Sigma) in a solution containing 0.15 M iron(iii) chloride hexahydrate (FeCl_3_, 97%, Alfa Aesar) and 1 M sodium nitrate (NaNO_3_, 99%, Alfa Aesar). The deposition was carried out at 100 °C for 1 h. After rinsing, the electrodes were annealed in a tube furnace at 800 °C for 5 minutes to convert β-FeOOH into hematite. For the rgH electrodes they were subjected to the same procedure one more time.

For aH (ALD) samples a Cambridge Nanotech, Savannah 100 atomic layer deposition apparatus was used. Iron *tert*-butoxide (heated to 125 °C) and water (25 °C) were used as the precursors for hematite and were pulsed alternatingly into the deposition chamber (heated to 180 °C) with a 10 cm^3^ min^–1^ flow of N_2_ as carrier gas. 500 cycles was used to produce films between 20–30 nm thick, grown onto FTO.^26^ Following the deposition, the samples were annealed at 500 °C for 15 minutes in air to assure all samples were completely converted into hematite.

### NiFeO_*x*_/GaO_*x*_ deposition

To deposit NiFeO_*x*_, Iron(iii) 2-ethylhexanoate (50% w/w in mineral spirits, Strem Chemicals) and nickel(ii) 2-ethylhexanoate (78% w/w in 2-ethylhexanoic acid, Strem Chemicals) were mixed in hexane to give a concentration of 15% w/w metal complex solution. The solution was further diluted 10 times with hexane and approximately 10 μL cm^–2^ of this solution was directly drop-casted onto the hematite electrodes. After drying in air for 5 minutes, the electrode was irradiated with UV light for 5 minutes.

GaO_*x*_ depositions were done following a previously reported procedure with slight adjustments.^42^ Hematite films were immersed in 50 mL of water at approximately 348 K. 0.209 g Ga(NO_3_)_3_·*n*H_2_O (99.9%, Aldrich) and 3.01 g of urea (99%, Aldrich) were added sequentially with an interval of 2 minutes under mild stirring. 20 min after the urea addition, the samples were rinsed with deionized water and annealed in a tube furnace at 1073 K for 5 minutes to improve crystallinity and stability.

### Characterization

To characterize the hematite films a Solartron Modulab XM potentiostat with a photoelectrochemical accessory was used for all current–voltage, IMPS, and IMVS measurements. Measurements were made in 1 M NaOH. A 3-electrode configuration was used with a platinum counter electrode, with a Hg/HgO reference electrode (CHI-152), and the hematite samples used as the working electrode. For the H_2_O_2_ study, 100 μL of 31 wt% H_2_O_2_ was added to 18 mL of 1 M NaOH. A 405 nm LED (ThorLabs) was used as the light source for all measurements, at 90% of its maximum power (134 mW cm^–2^). For IMPS and IMVS measurements a 10% modulation intensity was used, and the frequency was swept from 10 kHz down to 0.1 Hz. During the *JV* measurements a scan rate of 20 mV s^–1^ was used. All samples were measured using front side illumination.

## Supplementary Material

Supplementary information
