# Supplementary material for: Understanding the origin of photoelectrode performance enhancement by probing surface kinetics
Source: Chem Sci. 2016 Feb 11;7(5):3347–54. doi: 10.1039/c5sc04519c (PMC6006950; doi:10.1039/c5sc04519c)
Supplement: Supplementary file 1 [file SC-007-C5SC04519C-s001.pdf]

Electronic Supplementary Material (ESI) for Chemical Science.
This journal is © The Royal Society of Chemistry 2016

Supplemental Information

Understanding the Origin of Photoelectrode Performance Enhancement by Probing Surface Kinetics

James E. Thorne, Ji-Wook Jang, Erik Y. Liu, Dunwei Wang*

Department of Chemistry, Boston College, Merkert Chemistry Center, 2609 Beacon St., Chestnut Hill, MA 02467

*dunwei.wang@bc.edu

Current Voltage Curves

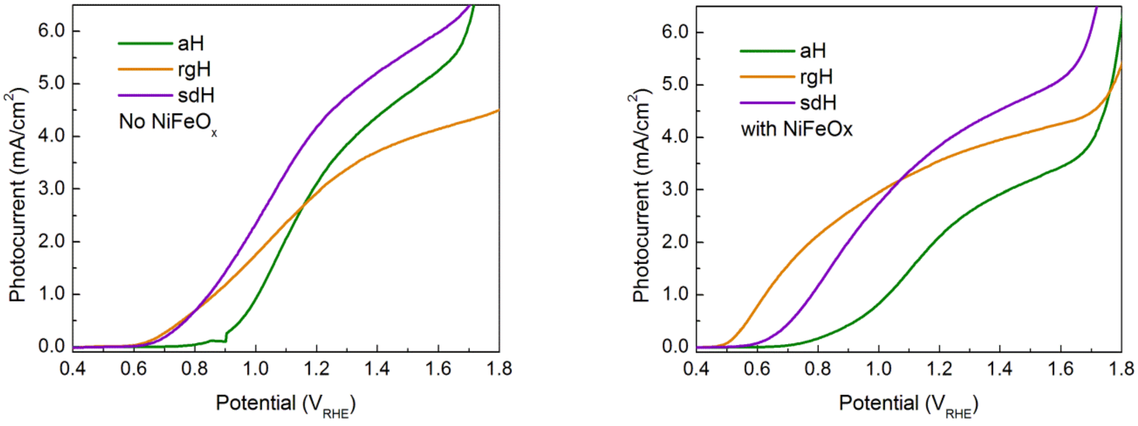

Figure S1. The JV curves shown are all under 405 nm light at 134 mW/cm^2. The left image shows the three hematite samples without $NiFeO_x$ on the surface. Where the right figure is after the $NiFeO_x$ has been added.

IMPS Fitting to Steady State Currents

Mott-Schottky analysis was used in order to determine the flat band potential and the doping density of aH electrodes. The measurements were made in the dark with a CH Instruments CHI604C potentiostat, in 1 M NaOH while stirring the solution. Both 100 Hz and 1000 Hz were sampled from 0.5 V to 1.1 V. The data set obtained was plotted using the following relation:

$$\frac{1}{C_S^2} = -\frac{N_D e}{2A^2 \varepsilon \varepsilon_0}\left[V_{sc} - V_{fb}\right] \qquad (1)$$

A linear line was found and the donor density of aH was determined to be 2.65 x 10^{19} cm^3. For the flat band potential a value of 0.73 V_{RHE} was obtained, consistent with other aH electrodes tested and previous values reported for aH.

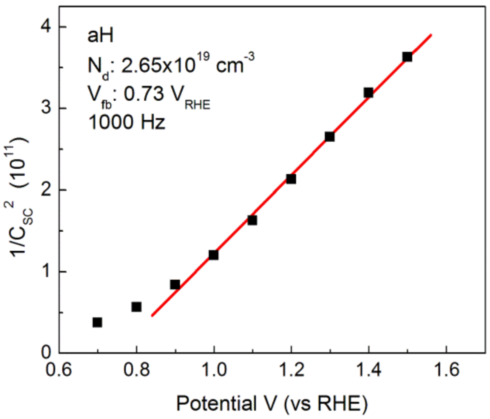

Figure S2. A Mott-Schottky plot of aH at 1000 Hz.

We next calculated the width of the space charge region using the following equation:

$$W_{sc} = \sqrt{\frac{2\epsilon\varepsilon_0(V_{app} - V_{fb})}{N_D q}} \qquad (2)$$

Where ε = 60, ε_0 = 8.85 x 10^{12} F/m, $V_{app} - V_{fb}$ is the magnitude of the space charge region assuming that all applied potential contributes to increased band bending, where V_{fb} = 0.73 V vs. RHE, and N_D = 2.65 x 10^{19} cm^{-3} for the aH sample used here. With the space charge region we were now able to fully calculate the Gartner current in our aH electrodes. Where the Gartner current is defined by:

$$J = qJ_0\left(1 - e^{\frac{\alpha W_{SC}}{1 + \alpha L_p}}\right) \qquad (3)$$

Here J is the Gartner current, J_0 = 2.2 x 10^{17} photons/cm^2 with a 405 nm LED (where the elementary charge, q, on an electron is used to convert the absorbed photons into an amperage), α is the absorption coefficient where a value of 1.00 x 10^5 cm^{-1} at 405 nm was used. A minority carrier length of 3 nm was employed here. After calculating the Gartner current we then measured the IMPS response of the same aH electrode in order to obtain the transfer efficiency of the electrode. For the electrode shown in the main text (fig 1) the values are shown in the table below.

Potential (V_{RHE})	Transfer Efficiency ($k_{tr}/(k_{tr} + k_{rec})$)
0.9	0.003
1	0.0314
1.1	0.302
1.2	0.746
1.3	0.899
1.4	0.961
1.5	0.973

Our initial steady state currents were cathodically shifted by ~100 mV. This mismatch in between the IMPS calculated steady state current and the dc steady state current has previously been reported.[1-2] Here we attribute this shift to the charging of the Helmholtz layer under steady state conditions that will be less prominent under ac incident lighting.

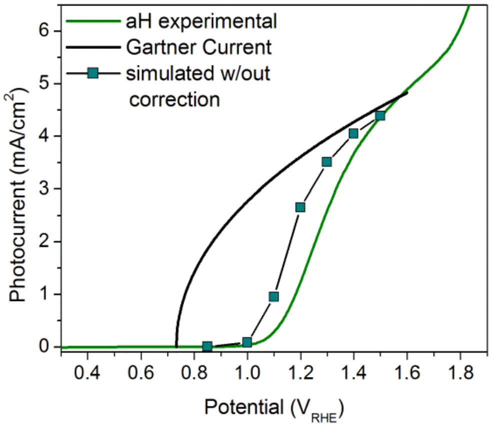

Figure S3. The Gartner current, aH experimental JV data, and the Gartner current multiplied by the transfer efficiency, not corrected for the potential drop across the Helmholtz layer.

In order to more closely fit our model, we next corrected for the potential drop across the Helmholtz layer across the studied potential range, 0.5 – 1.2 V vs. RHE. This can be done by calculating the change in potential across the Helmholtz layer with the following equation:

$$\Delta V = \frac{qp_{surf}}{C_H + C_{SC}} \qquad (4)$$

Where q is the elementary charge, C_H is the Helmholtz capacitance, 100 μF was used here. C_{SC} is the space charge capacitance, as taken from the Mott-Schottky analysis and was on the order of 0.1-0.3 μF. Lastly, p_{surf} is the surface concentration of holes. In order to calculate the surface concentration of holes, the gartner current is divided by the recombination and transfer rates, as shown below.

$$p_{surf} = \frac{J}{k_{tran} + k_{rec}} \qquad (5)$$

J here is the calculated Gartner current, and $k_{tran} + k_{rec}$ is determined by the apex of the upper quadrant IMPS semicircle, as seen in Fig1a in the main text. Ultimately a surface hole concentration of 1-10 x 10^{13} holes/cm^2 was determined. After plugging all of the values into equation 4 and 5 the light induced potential drop across the Helmholtz layer was determined. For the aH sample shown in the text, the table below shows the determined values.

**Potential (V_{RHE})**	**ΔV across Helmholtz layer**
0.9	0.0343
1.0	0.107
1.1	0.126
1.2	0.112
1.3	0.124
1.4	0.168

When applying the potential drop across the Helmholtz layer to the original Gartner + transfer efficiency, the fit becomes much closer to the experimental values that were observed for the same aH electrode. This analysis was limited to aH electrodes, since the Mott-Schottky analysis is less straight forward for nanostructured materials.

IMPS Measurements

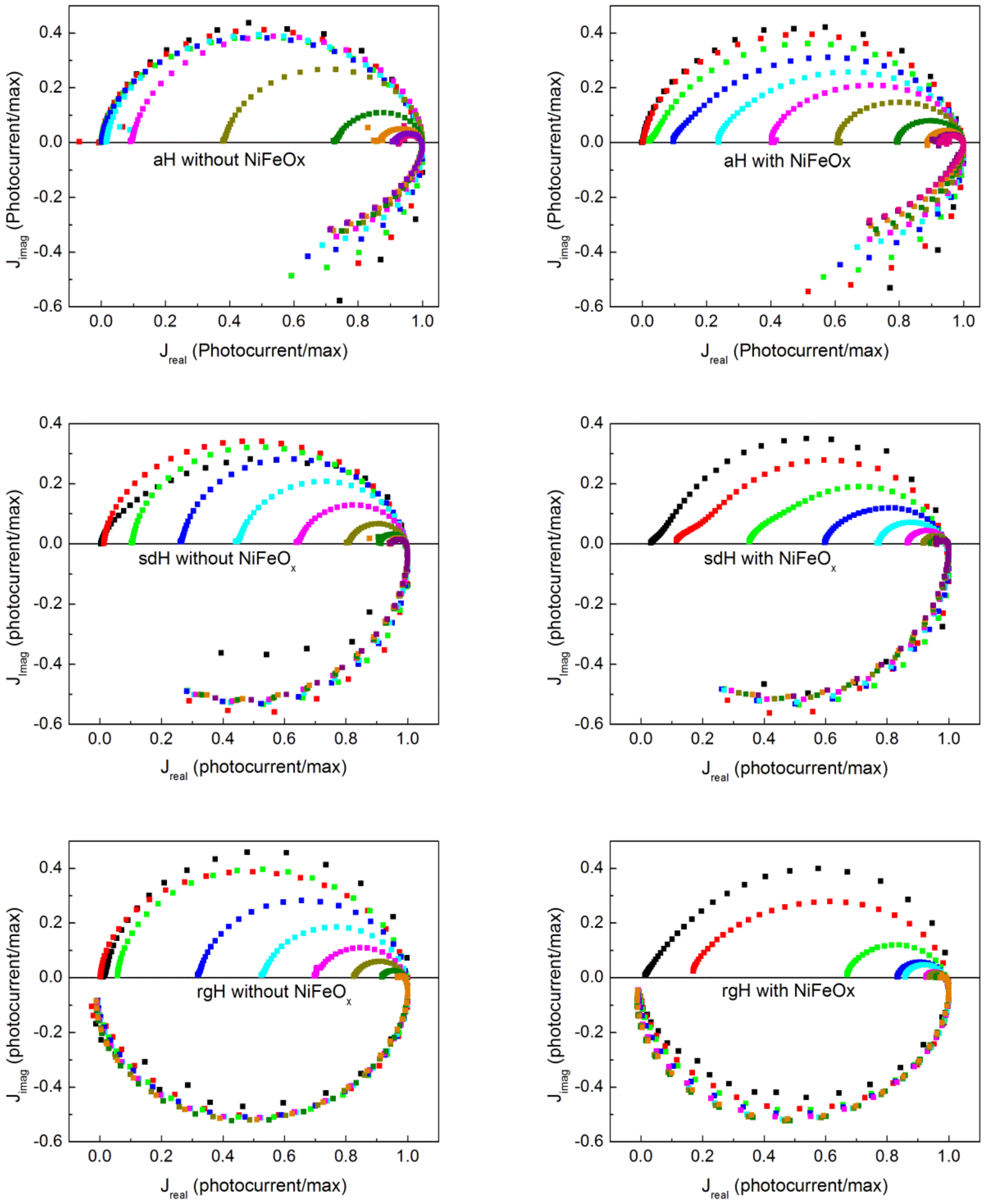

Figure S4. IMPS measurements of all six samples tested for this study. The high frequency crossing point with the real photocurrent axis was used to normalize the imaginary and real axes. This allows for an easy comparison between samples at different applied potential, and for the transfer efficiency to be easily determined. The black trace represents a 0.4 V_{RHE} applied potential for each graph with 0.1 V steps up to 1.4 V_{RHE}. The order of the traces goes, black, red, green, blue, cyan, magenta, dark yellow, olive green, orange, purple, pink. Each color represents the same applied potential in each graph.

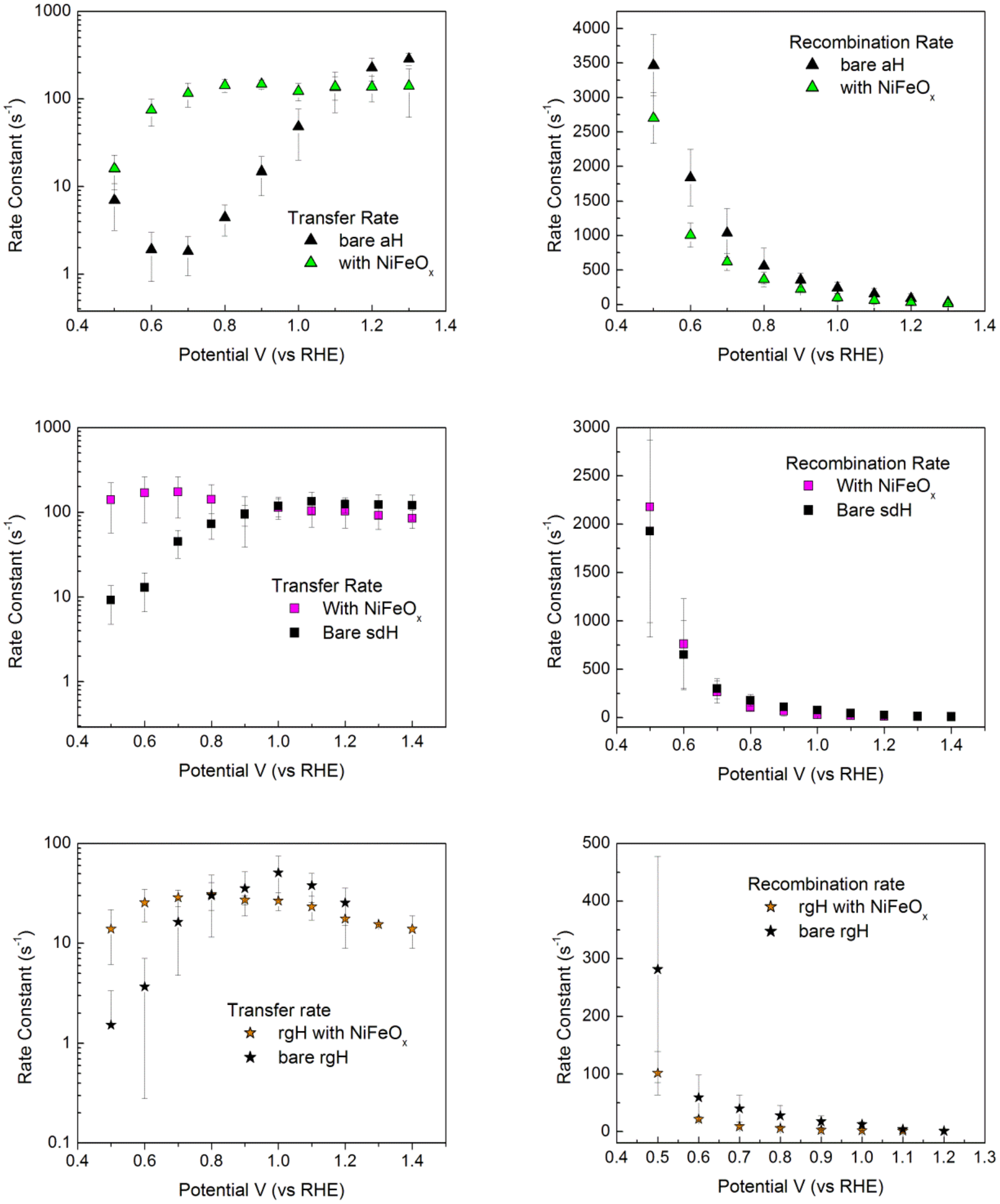

Figure S5. k_{tran} and k_{rec} plotted versus the applied potential as determined by IMPS. Triangles are used for the aH samples, with bright green used at the trace with $NiFeO_x$ added. Squares are used for sdH and purple is used for the samples with $NiFeO_x$. Stars are used for rgH with orange showing the samples with $NiFeO_x$.

	Water Oxidation Rate Constant					
	aldH		sdH		rgH2	
V_{RHE}	No $NiFeO_x$ (s^{-1})	With $NiFeO_x$ (s^{-1})	No $NiFeO_x$ (s^{-1})	With $NiFeO_x$ (s^{-1})	No $NiFeO_x$ (s^{-1})	With $NiFeO_x$ (s^{-1})
**0.5**	6.97	15.9	9.21	140	1.52	13.9
**0.6**	1.91	74.5	12.9	168	3.66	25.5
**0.7**	1.82	116	44.9	174	16.2	28.8
**0.8**	4.44	143	72.4	141	30.0	30.9
**0.9**	14.8	147	94.1	95.6	35.4	27.2
**1.0**	48.2	122	118	113	50.9	26.6
**1.1**	135	137	133	103	37.8	23.2
**1.2**	226	136	123	102	25.4	17.5
**1.3**	286	140	122	91.3	19.6	15.5

Table S1. The numerical values obtained from k_{tran} values in figure S2. The blue shaded regions show where the k_{tran} values are higher for the sample without $NiFeO_x$ than with $NiFeO_x$.

IMPS at Varying Light Intensities

At lower light intensities it is expected that the quasi Fermi level splitting in light will be reduced. This will lead to lower rate constants in a system with Fermi level pinning. In addition, since the band bending will be determined by the applied potential, the surface recombination should remain constant. Figure S6 shows that this is indeed the case, with figure S7 providing a schematic of the system at different light intensities.

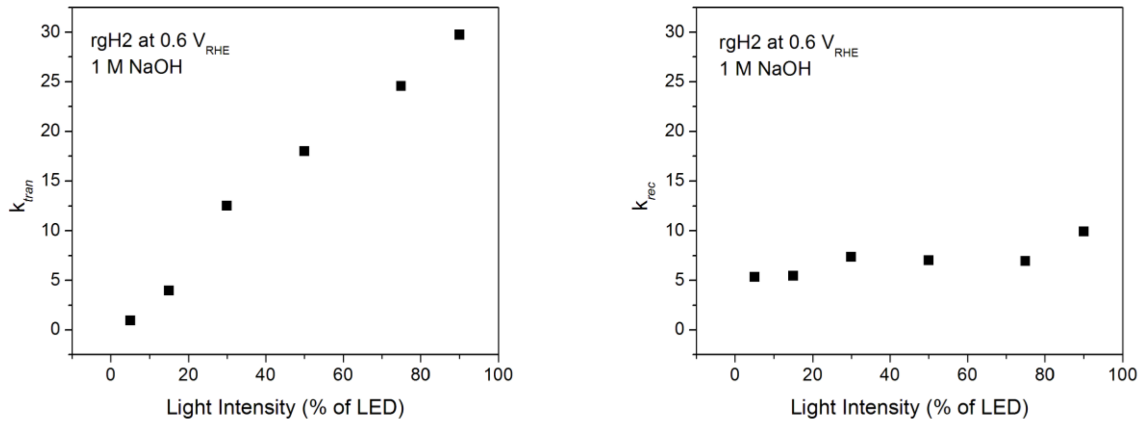

Figure S6. IMPS measurements made at 5 – 90% of the LED intensity, where 90% is 134 mW/cm^2. The samples tested here are bare electrodes. The figure on the left shows the transfer rate constant, which increases monotonically with light intensity. The figure on the right shows the recombination which remains nearly constant at all light intensities probed. All measurements were made on rgh2 at 0.6 V vs RHE, which is slightly after the onset of the photocurrent for these photoelectrodes.

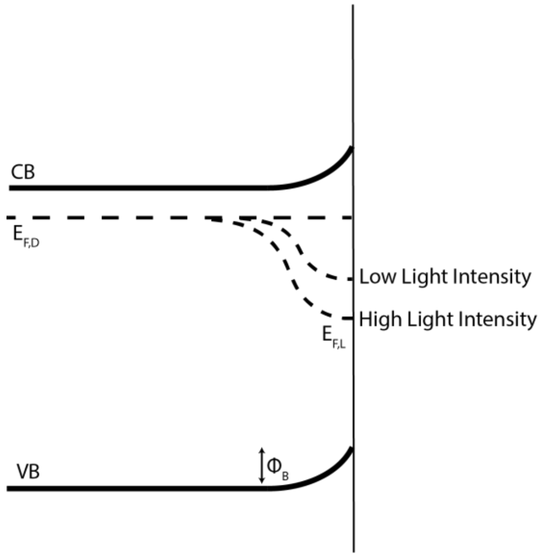

Figure S7: A schematic of the bands with different light intensities. When the light intensity is greater, the Fermi level splitting is greater, reducing the activation energy for water oxidation, as seen in Figure S6. The applied potential for the low intensity and high intensity is the same, resulting in same degree of band bending (Φ_B), resulting in similar rate constants for recombination, also as seen in Figure S6. Here, E_{FL} and E_{FD} correspond to the quasi Fermi level in light and the Fermi level in dark, respectively.

IMPS with and without H_2O_2

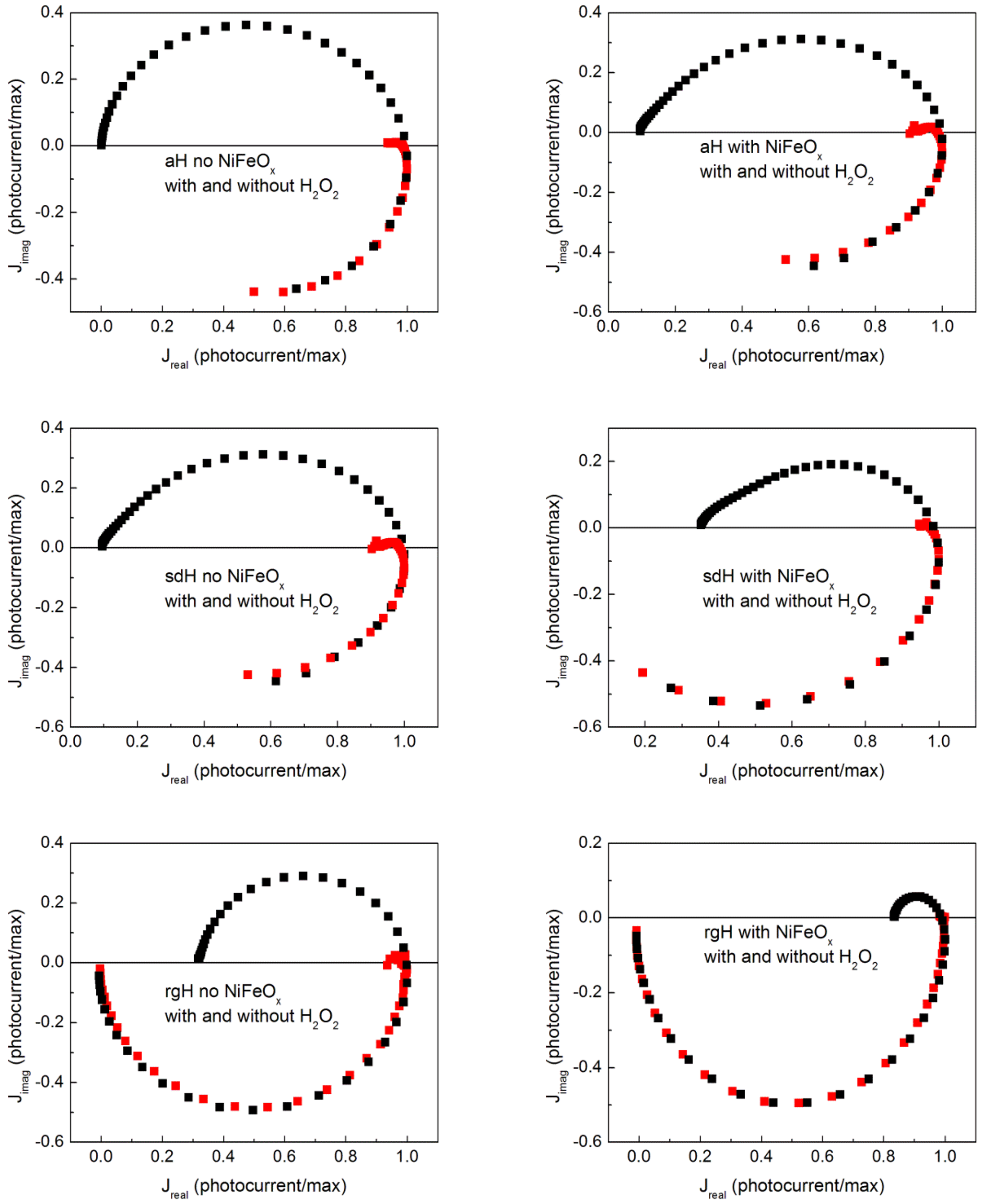

Figure S8. All IMPS measurements were made at 0.7 V_{RHE}. The black trace is without H_2O_2 and the red trace is with H_2O_2. The figures on the right are with $NiFeO_x$ and the figures on the left are without $NiFeO_x$. Especially with aH, it is seen that the addition of H_2O_2 takes the transfer efficiency to near unity well before any onset potential is observed without the H_2O_2.

References:

(1) de Wit, A. R.; Vanmaekelbergh, D.; Kelly, J. J., A Study of the Photoanodic Dissolution of CdS with Electrical and Opto-electrical Impedance Spectroscopy. *J. Electrochem. Soc.* **1992,** *139*, 2508-2513.

(2) Peter, L. M.; Ponomarev, E. A.; Fermín, D. J., Intensity-modulated photocurrent spectroscopy: reconciliation of phenomenological analysis with multistep electron transfer mechanisms. *J. Electroanal. Chem.* **1997,** *427*, 79-96.
